# Role of Toll-Interacting Protein Gene Polymorphisms in Leprosy Mexican Patients

**DOI:** 10.1155/2013/459169

**Published:** 2013-11-04

**Authors:** Margarita Montoya-Buelna, Mary Fafutis-Morris, Alvaro J. Tovar-Cuevas, Anabell Alvarado-Navarro, Yeminia Valle, Jorge R. Padilla-Gutierrez, Jose F. Muñoz-Valle, Luis E. Figuera-Villanueva

**Affiliations:** ^1^Centro Universitario de Tonalá, Universidad de Guadalajara, Tonalá, Jal., Mexico; ^2^Centro Universitario de Ciencias de la Salud, Universidad de Guadalajara, Guadalajara, Jal., Mexico; ^3^CIINDE Universidad de Guadalajara/Instituto Dermatológico de Jalisco “Dr. Jose Barba Rubio”, Sierra Mojada 950, Edificio P. Primer Nivel, Colonia Independencia, 44340 Guadalajara, Jal., Mexico; ^4^Genetic Division Department, Biomedical Research Center West, Instituto Mexicano del Seguro Social, Sierra Mojada 800, Colonia Independencia, 44340 Guadalajara, Jal., Mexico

## Abstract

*Background*. Leprosy is a debilitating infectious disease of human skin and nerves. Genetics factors of the host play an important role in the disease susceptibility. Toll-interacting protein (TOLLIP) is an inhibitory adaptor protein within the toll-like receptor (TLR) pathway, which recognizes structurally conserved molecular patterns of microbial pathogens, initiating immune responses. The objective of this study was to investigate the association of variants in the *TOLLIP* gene with susceptibility to leprosy in Mexican patients. *Methods*. *TOLLIP* polymorphisms were studied using a case-control design of Mexican patients with lepromatous leprosy (LL). The polymorphisms of *TOLLIP* at loci −526 C>G (rs5743854), 1309956C>T (rs3750920), 1298430C>A (rs5744015), and 1292831 G>A (rs3750919) were analyzed by PCR, with sequence-specific primers in LL patients and healthy subjects (HS) as controls. *Results*. Genotype distributions were in Hardy Weinberg equilibrium for all sites except for rs3750920. Neither genotype nor allele frequencies were statistically different between LL patients and controls (*P* > 0.05). The maximum pairwise D' coefficient reached was 0.44 of linkage (*P* = 0.01) for all the polymorphisms except for rs5743854. The three loci haplotype comparison yielded no significant differences between groups. *Conclusions*. Just the individuals with genotype C/C of rs3750920 have a trend of protective effect to developing LL.

## 1. Background

Leprosy (L), or Hansen's disease, is a chronic infection caused by the intracellular pathogen *Mycobacterium leprae *[1]. The registered prevalence globally at the beginning of 2012 was 181,941 leprosy patients [2]. Leprosy is a disease that presents as a spectrum of clinical manifestations that depend on the host cell-mediated immune response against the *M. leprae *[3]. One end of the disease spectrum, patients with tuberculoid leprosy (TT), shows a strong cellular immune response, resulting in resistance responses that restrict the growth of the pathogen. The number of lesions is few and bacilli rare, although tissue and nerve damage are frequent. At the opposite end of this spectrum, patients with lepromatous leprosy (LL) have a limited cellular immune response, making them susceptible to disseminated infection. Skin lesions are numerous and growth of the pathogen is unabated. The factors that influence which type of leprosy develops are not well understood. Many epidemiological studies, which had the objective of identifying susceptibility genes [4], have shown how host genetic factors play a role in the variability of clinical response to infection caused by *M. leprae*.

Human toll-like receptors (TLRs) are a family of pattern recognition receptors capable of initiating innate immune responses and influencing subsequent adaptive immune responses [5]. Mycobacterial TLR ligands include molecules of bacterial cell wall components, like that found in *M. leprae *bacilli (lipomannan, lipoarabinomannan, phosphatidylinositol-dimannoside, and a 19-kDa lipoprotein) [6–11]. Their activation in response to microbial infection and inflammation triggers NF-*κ*B and mitogen-activated proteins kinase (MAPK) signaling and culminates in the induction of host defense genes, including the production of numerous cytokines, chemokines, adhesion molecules, and enzymes. However, excessive and prolonged activation of innate immunity is harmful to the host and may even be fatal [12]. Signaling must therefore be tightly controlled to ensure a beneficial response with the appropriate magnitude and duration. Multiple mechanisms exist to halt IL1RI and TLR signaling pathways, including the activity of several inducible signaling suppressors (i.e., MyD88 and IRAK-M). Toll-interacting protein (TOLLIP) is an adaptor protein and acts as an inhibitory factor in the TLR-signaling cascade; overexpression of TOLLIP impairs IL1RI-, TLR2-, and TLR4-triggered NF-*κ*B and JNK signaling pathways [12–15]. Following the stimulation of these receptors, TOLLIP associates directly with the cytoplasmic TIR domain [12, 16], suppressing the phosphorylation and kinase activity of interleukin-1 receptor associated kinase 1 (IRAK-1) [16], and in this way controls the magnitude of inflammatory response to endotoxin [17].

TOLLIP is therefore thought to function mainly to maintain immune cells in a quiescent state and to facilitate the termination of IL-1R/TLR-induced cell signaling during inflammation via suppression of IRAK-1's activity [13, 16]. TOLLIP, moreover, deregulated inhibition of this pathway might result in attenuated responses to the proinflammatory cytokines (IL-6 and TNF-*α*) suppression of TLR-mediated cellular responses and a role in the leprosy.

Several single-nucleotide polymorphisms in the TLR signaling pathways and their negative regulators genes have been reported to influence the production of inflammatory cytokines and implicated in the pathogenesis of allergic, autoimmune as well as to infectious diseases [18–26]. However, effects of genetic variation in TLRs downstream signaling pathways have not been amply studied yet.

The *TOLLIP *gene is located on chromosome 11p15 (MIM: 606 277) and comprises 6 exon encoding 274 amino acid transcripts. It is a negative regulator of TLR signaling cascade and was implicated in suppression of the TLR2 [12] and TLR4 pathways [14]. We compared the distribution of four SNPs at *TOLLIP *gene: −526 C/G (rs5743854), exon 4 Pro139Pro (rs3750920), exon 6 Ala222Ser (rs5744015), and 3′UTR (rs3750919) in a population-based design study of LL Mexican patients.

## 2. Methods

### 2.1. Patients and Controls

Leprosy patients (*n* = 93) attended in the Instituto Dermatologico de Jalisco (SSA) and took part in this study. All patients were residents in the western of Mexico. They were classified according to clinical and laboratorial (Mitsuda's test and histology exams) observations made by dermatologist responsible for leprosy diagnosis as LL patients, summarized in Table 1.

The control group was composed of 152 healthy individuals of Mexican mestizo ethnicity, selected according to demographic parameters. The mestizo ethnicity is the result of genetic crossing between Spanish and Amerindian people [27] and represents 80% of the overall population of Mexico [28]. Matching patients minimized the risk of population stratification bias, due to differences in ethnic background between patients and controls, and variations of allele frequencies, according to ethnic background, with control individuals of the same ethnic backgrounds and residence in the same geographical areas of leprosy prevalence. Ethics committee of the Instituto Dermatologico de Jalisco (SSA) approved the study and Declarations of Helsinki protocols were followed. Samples were taken only after participants voluntarily signed an informed consent form. Blood samples were collected from patients and healthy subjects (HS) in ethylenediamine tetra-acetic acid anticoagulant. DNA was extracted using a standard salting-out method [29].

### 2.2. Genotyping

All four polymorphisms in the *TOLLIP *gene were genotyped by restriction enzyme digestion (see Table 1). Patient and control samples were amplified using the same primers and conditions as previously described by Schimming et al. [30]. After digestion with the respective enzymes for at least three hours, the fragments were separated on polyacrylamide gel, electrophoresed in 1X TBE buffer (Native PAGE), and viewed by staining with 0.2% silver nitrate according to Sanguinetti et al. [31].

### 2.3. Statistical Analysis

Allele frequency was obtained by direct counting. The agreement of Hardy-Weinberg equilibrium was evaluated using the SAS software v8 (SAS Institute Inc., Cary, NC, USA). Statistical significance was indicated by *P* < 0.05. The odds ratios (OR), with 95% confidence intervals (95% CI), were calculated using SISA statistics online (http://home.clara.net/sisa/), to evaluate the risk of the individual developing the disease while having a particular *TOLLIP *type. Haplotypes frequencies were inferred using EMHAPFREQ software [32]. Pairwise linkage disequilibrium (LD) was expressed as Lewontin's D′ corrected coefficient [33]. Comparison data was evaluated by *χ* ^2^ test or the Fisher exact-test when applicable.

## 3. Results

A total of 98 LL patients were recruited and 152 unrelated otherwise healthy individual as a control. Analysis of genotype and allele frequencies of *TOLLIP *was done in both groups, summarized in Table 2. In controls genotype distributions were in Hardy Weinberg equilibrium for all sites except for rs3750920 (exon 4).

### 3.1. Leprosy Susceptibility

To identify a genetic relation between *TOLLIP *SNPs and lepromatous pole of clinical spectrum of leprosy, four previously published *TOLLIP *polymorphisms at loci −526 C>G (rs5743854), 1309956C>T (rs3750920), 1298430C>A (rs5744015), and 1292831 G>A (rs3750919) were screened [30]. Our results showed that the genotypes frequencies of tagging SNPs rs5743854 (*P* = 0.76), rs3750920 (*P* = 0.05), rs5744015 (*P* = 0.76), and rs3750919 (*P* = 0.76) were nonsignificant differences of LL patients *versus *HS. However, in LL patients a decreased frequency in C/C genotype of rs3750920 (25%), in comparison with HS (37%), was observed (*P* = 0.05). These trends could suggest a protective effect in western population from Mexico. Also, when we analyzed the allele frequencies no significant differences were found (rs5743854, *P* = 0.15; rs3750920, *P* = 0.09; rs5744015, *P* = 1.00; rs3750919, *P* = 0.29). The allele and genotype frequencies of the four tagging SNPs in the LL patients and control subjects and the statistical analysis results are listed in Table 2.

### 3.2. Haplotype and Linkage Disequilibrium Estimation

Haplotypes were estimated based on the genotype frequencies of the LL patients and control group (Table 3). Regarding to linkage disequilibrium analysis (Table 3), only the polymorphisms located at exon 4, exon 6, and 3′UTR showed nonrandom segregation, where the maximum D′ coefficient reached a moderate value of LD (LD = 0.44, Bonferroni correction *P* value = 0.01) [34]. The haplotype comparison for these linked sites (rs3750920, rs5744015, and rs3750919) showed similar distributions between LL patients and the control group. Conversely, Schimming et al. [30] found linkage evidence between the promoter (rs5743854) and 3′UTR (rs3750919) and other intermediate polymorphisms. Since the chromosomal position of the polymorphisms was tested in this study, we expected similar results; however as it has been described LD is influenced by population type [35] among other factors [34]. 

## 4. Discussion

In leprosy, a variety of host immune-genetic factors seem to influence subjects' susceptibility to *M. leprae *and disease course. Human regulation and nature of an optimal inflammatory response to *M. leprae *remain poorly understood. Mycobacterial cell wall contains a number of toll-like receptors (TLRs) ligands, including lipoproteins, mycolylarabinogalactan-peptidoglycan complexes, lipids, and lipoarabinomannan (LAM). Although TLRs are crucial for host defense, it has become apparent that loss of negative regulation of TLR signaling is strongly linked to acute and chronic inflammation, as well as systemic autoimmune diseases [18–25]. In leprosy patients, TLR activation may promote excessive inflammation and apoptosis contributing to pathological signs such as nerve damage [36]. A balance of TLR expression and activation could be crucial to avoid an excessive inflammatory response and lessen disease severity. Negative regulation of TLR-induced response is important for suppressing inflammation and deleterious immune responses. TOLLIP has been identified as a negative regulator that suppresses TLR signaling at multiple levels, and thus high levels could diminish TLR signaling; this phenomenon has been observed in response to pathogen challenge [13, 37, 38]. Some bacterial components stimulate TLR signaling, activating NF*κ*B, JNK, and p38 signaling cascades, leading to transcription and translation of IL-6 and TNF*α* [39]. Thus, beneficial or deleterious effects of TOLLIP in leprosy patients depend on expression level and time of exposure to proinflammatory cytokine. It is hypothesized that in leprosy patients high TOLLIP levels could generate a low inflammatory response against mycobacteria; however low TOLLIP levels could produce a sustained inflammatory response which is associated with tissue damage. Screening of *TOLLIP* gene by sequencing and SSCP analysis [26, 30, 40] has shown several mutations, which affect gene expression and biological function modifying inflammatory-cytokine production. Therefore, we evaluated whether some of these mutations could influence the susceptibility to LL. We analyze four *TOLLIP* SNPs, rs5743854 at promoter region, rs3750920 and rs5744015 in the coding region, and rs3750919 at 3′UTR.

Our study shows that there is no significant association between genotype and allele frequencies of *TOLLIP* variation −526 G>C (rs5743854) with LL (Table 2). However it has been observed that the same variation of *TOLLIP* gene showed borderline association with atopic dermatitis, which is a common chronic inflammatory skin disorder. We observed in healthy heterozygous subjects that genotype rs5743854 G/C showed a similar frequency (14%) to that reported in Caucasian German control subjects (15.4%) [30]. Furthermore, allele frequency in our control group (C allele 90%, A allele 10%) showed a similar allele frequency compared to German controls (C allele 92.3%, A allele 7.7%).

We observed that rs3750920 C/T genotype was predominant in LL patients (64%) and controls (54%); compared to C/C (LL patients (25%) and controls (37%)) and T/T (LL patients (11%) and controls (9%)) genotypes. Allele C frequency was also more common in LL (57%) and controls (63%) groups. Moreover, LL patients showed a decreased frequency in rs3750920 C/C genotype (25%), in comparison to HS (37%) (*P* = 0.05). These tendencies suggest a protective effect to eventually development LL disease (OR 1.77) in Mexican western population. However Shah et al. [26] observed that rs3750920 SNPs were associated with protection from tuberculosis and increased levels of *TOLLIP* mRNA, and this was the first association of *TOLLIP* polymorphisms with any infectious disease. These differences could be due to different genetic backgrounds (Amerindians, Spanish, and West African, predominantly) from analyzed subjects, which have different admixture grades depending on Mexico region. Therefore, this genetic heterogeneity (also described as asymmetric admixture) could explain intra- and interpopulation differences in significant gene-diseases associations and prevalence for some diseases. Although the best solution to describe these associations would be the analysis of admixture informative markers (AIMs) to probe the ancestral homogeneity between case and control population samples, a practical solution could be done to demonstrate that the locus of interest has similar allele frequency in parental populations, avoiding population structures problems to achieve convincing inferences.

We also evaluated rs5744015 and rs3750919 polymorphisms variants, and none of them showed significant differences in allelic and genotypic frequencies from patients versus control group. We observed that rs5744015 C/C genotype was predominant in LL patients (98%) and controls (97%), compared to C/A (LL patients (2%) and controls (3%)) and A/A (LL patients (0%) and controls (0%)) genotypes. Allele C frequency was also more common in LL (99%) and control (99%) groups. The A/A genotype was not present in patients and controls, which correlates with results found in studies [30] where A/A genotype was also absent in patients and present in 0.5% in subjects from the control group. Finally for rs3750919 region, G/G genotype was predominant in LL patients (49%) and controls (53%), compared to G/A (LL patients (41%) and controls (41%)) and A/A (LL patients (10%) and controls (6%)) genotypes. Allele G frequency was also more common in LL (69%) and control (74%) groups. Therefore, G/G genotype frequencies as G allele were the most common in our patients and controls, and these observations were similar to those observed in previous reports on patients with atopic dermatitis [30]. Exhaustive sequencing is required to find or rule out the possibility of an as-yet-unknown causal SNP in LL with rs5744015 and rs3750919. Further functional evaluation of novel or associated SNPs is also needed. 

Concerning to linkage disequilibrium analysis (Table 3), only the polymorphisms located at exon 4, exon 6, and 3′UTR showed nonrandom segregation [34]. The haplotype comparison for these linked sites (rs3750920, rs5744015, and rs3750919) showed similar distributions between LL patients and control group. Therefore even when alleles show linkage disequilibrium this circumstance has no association with risk to develop leprosy in Mexican patients.

The reasons for nonreplication of association studies are numerous and largely reflect inadequate sample sizes, although differences may also be attributed to population stratification, variation in study design, confounding sampling bias, and missclassification of phenotypes. We cannot exclude the possibility that the association result founded for *TOLLIP *genotype frequencies with LL could be related to other *TOLLIP *SNPs or neighboring genes not evaluated in this study, which presents LD with this variation. As additional studies, we contemplate including more patients, evaluating cytokine levels (IL-10, TNF-*α*, and IL-6), and mRNA expression of *TOLLIP*.

Taken together, these findings represent new insights for a better comprehension of negative regulation of TLR4 and TLR 2 signaling pathway and effective mechanisms for therapeutic intervention and treatment of inflammatory diseases. We need to consider the recent and impacting findings reported by Masaki et al., in their *in vitro* studies where they demonstrate that *M. leprae* is able to induce cellular reprogramming though epigenetics changes, which take part in the migration, plasticity, and immunomodulatory functions, which are finally employed by *M. leprae* to avoid the immune response [41].

## 5. Conclusion

Individuals with genotype C/C of rs3750920 have a trend of protective effect to developing LL, and rs5743854, rs5744015, and rs3750919 SNPs are not associated with LL. In the global test haplotypes analyses were not associated with LL risk in Mexican patients. Genetic analysis of genes involved in susceptibility to leprosy phenotypes will give us the clues of genetic mechanisms from Mexican leprosy patients and other ethnic or racial backgrounds.

## Figures and Tables

**Table 1:** Clinical and demographic characteristics of patients with LL.

Characteristic	LL *n* = 98
Disease duration (years), mean ± SD	10 ± 7.9
Age, mean ± SD	54.63 ± 15.92
Gender	
Female (%)	36
Male (%)	62
Family history (%)	38
Bacilloscopy (%, ++/+++)	100
Mitsuda test (%, negatives)	100
Treatment PCT (%)	56
New cases (non-Tx, %)	44

Quantitative variables are expressed as means ± standard deviations (SD) and qualitative variables as frequencies and percentages (%) as noted. LL: lepromatous leprosy. PCT: polychemotherapy (Rifampin, Clofazimine, Dapsone). Tx: treatment. Family history refers to at least one first grade family who has been infected with leprosy bacillus. Bacilloscopy samples were taken from ear smear.

**Table 2:** Genotype and allele distributions of −526, exon 4, exon 6, and* 3*′*UTR TOLLIP *polymorphisms in LL and HS.

Polymorphism	LL (*n* = 98) *n* (%)	HS (*n* = 152) *n* (%)	OR CI (95%)	*P* value
rs5743854				
(−526 C>G)				
Genotype				
C/C	84 (86)	127 (84)		
C/G	13 (13)	22 (14)	0.89 (0.43–1.87)	*P* = 0.76
G/G	1 (1)	3 (2)	0.50 (0.05–4.93)	*P* = 0.55
Allele				
C	181 (92)	276 (91)		
G	15 (8)	28 (9)	0.82 (0.42–0.16)	*P* = 0.54
* HWE = 0.43 *				
rs3750920				
(1309956 C>T)				
Genotype				
C/C	24 (25)	56 (37)		
C/T	63 (64)	83 (54)	1.77 (0.99–3.16)	*P* = 0.05
T/T	11 (11)	13 (9)	1.97 (0.772–5.02)	*P* = 0.15
Allele				
C	111 (57)	195 (64)		
T	85 (43)	109 (36)	1.37 (0.95–1.98)	*P* = 0.09
*HWE = 0.04 *				
rs5744015				
(1298430 C>A)				
Genotype				
C/C	96 (98)	148 (97)		
C/A	2 (2)	4 (3)	0.80 (0.14–4.29)	*P* = 0.76
A/A	0 (0)	0 (0)	1.54 (0.03–78.2)	*P* = 1.00
Allele				
C	194 (99)	300 (99)		
A	2 (1)	4 (1)	0.77 (0.14–4.26)	*P* = 1.00
*HWE = 1.00 *				
rs3750919				
(1292831 G>A)				
Genotype				
G/G	48 (49)	81 (53)		
G/A	40 (41)	62 (41)	1.09 (0.64–1.86)	*P* = 0.76
A/A	10 (10)	9 (6)	1.87 (0.71–4.94)	*P* = 0.19
Allele				
G	136 (69)	224 (74)		
A	60 (31)	80 (26)	1.24 (0.83–1.84)	*P* = 0.29
*HWE = 0.81 *				

LL: lepromatous leprosy; HS: healthy subjects; OR: odds ratio; C: confidence interval; HWE: Hardy-Weinberg equilibrium *P* value; codominant model is presented.

**Table 3:** Haplotype frequencies of the rs3750920, rs5744015, and rs3750919 *TOLLIP *polymorphisms in LL and HS.

Haplotype	LL (2*n* = 196) *n* (%)	HS (2*n* = 304) *n* (%)	OR CI (95%)	*P* value
CCG	96 (48.9)	160 (52.7)	*	
CCA	15 (7.68)	32 (10.5)	0.78 (0.40–1.52)	*P* = 0.47
CAA	0 (0)	1 (0.002)	0.55 (0.02–13.7)	*P* = 0.72
CAG	0 (0)	2 (0.008)	0.33 (0.02–7.00)	*P* = 0.48
TCG	38 (19.4)	61 (20.2)	1.03 (0.64–1.67)	*P* = 0.88
TCA	45 (22.9)	47 (15.4)	1.59 (0.99–2.58)	*P* = 0.06
TAA	0 (0)	1 (0.002)	1.66 (0.03–84.5)	*P* = 0.80
TAG	2 (1.02)	0 (0)	8.3 (0.40–175)	*P* = 0.17

LL: lepromatous leprosy; HS: healthy subjects; OR: odds ratio; CI: confidence interval; *Reference haplotype.

## Abbreviations

CI: Confidence interval
HS: Healthy subjects
IRAK-1: Interleukin-1 receptor associated kinase 1
L: Leprosy
LD: Linkage disequilibrium
LL: Lepromatous leprosy
OR: Odds ratio
PAMP: Pathogen-associated molecular pattern
PCR: Polymerase chain reaction
RFLP: Restriction fragment length polymorphisms
SNP: Single nucleotide polymorphisms
TLR: Toll-like receptor
TOLLIP: Toll-interacting protein
TT: Tuberculoid leprosy
*M. leprae*: *Mycobacterium leprae*.
